# Influence of HLB Value of Emulsifier on the Properties of Microcapsules and Self-Healing Properties of Waterborne Coatings

**DOI:** 10.3390/polym14071304

**Published:** 2022-03-24

**Authors:** Yu Tao, Xiaoxing Yan

**Affiliations:** 1 Co-Innovation Center of Efficient Processing and Utilization of Forest Resources, Nanjing Forestry University, Nanjing 210037, China; taoyu@njfu.edu.cn; 2 College of Furnishings and Industrial Design, Nanjing Forestry University, Nanjing 210037, China

**Keywords:** self-healing microcapsules, waterborne coatings, HLB value

## Abstract

In this paper, self-healing microcapsules were prepared by using melamine–formaldehyde (MF) resin as the wall material and shellac as the core material repairing agent. In order to explore the effect of the four factors (i.e., the HLB value of emulsifier, the type of solvent, the mass ratio of shellac to rosin, and the rate of rotating) on the comprehensive performance of microcapsules, and orthogonal experiments with four factors and three levels were carried out. The results showed that the hydrophilic lipophilic balance (HLB) value of the emulsifier was the most important influencing factor. In order to further explore the relationship between the HLB value of the emulsifier and the morphology of the microcapsules and the coating rate as well as to further optimize the performance of the microcapsules, taking the HLB value of the emulsifier as the single factor variable, single-factor tests were carried out. The results showed that when the HLB value was 12.56, the microcapsules of melamine–formaldehyde resin-coated shellac–rosin mixture had a uniform distribution and high coating rate. In order to explore the self-healing properties of waterborne coatings with microcapsules, the microcapsules prepared by single-factor experiments were mixed into the waterborne coatings at mass ratios of 0%, 3.0%, 6.0%, 9.0%, 12.0%, and 15.0%. It showed that the elongation at break of the waterborne coating with the addition of 3.0% microcapsule at mass fraction was improved, and it had a higher repair rate. This study provides a new research idea for the optimization and characterization of the self-healing properties of waterborne coatings.

## 1. Introduction

The coating on the surface of an object has the functions of decoration, isolation, and protection [1,2]. Once cracks are formed in a coating, its performance and the protective effect on the substrate will decline [3,4]. Therefore, maintaining the original properties of the coating is a key factor in prolonging the role of the coating as a protective layer. In order to achieve this goal, scholars have explored self-healing coatings that can automatically repair damaged areas [5,6]. Adding microcapsules containing a core repair agent to the coating is one of the most studied methods at present [7]. In this case, when the material is cracked, the microcapsules containing the repairing agent are ruptured, and the repairing agent is released at the damaged part, which reacts with the pre-embedded catalyst to synthesize a new polymer, thereby realizing the function of self-healing [8,9]. This allows the coating to continue to perform its function of protecting the substrate [10].

Yin et al. [11] synthesized urea formaldehyde-coated epoxy resin microcapsules and a two-component self-healing system with CuBr_2_(2-meim)_4_ as latent curing agent. However, the system had a strong dependence on temperature, and the self-healing curing reaction could not occur at room temperature. Coope et al. [12] explored a novel Lewis acid catalyzed self-healing system and studied the effects of microcapsule content, self-healing temperature, and time on the self-healing performance of the system. The self-healing performance was quantitatively analyzed using a conical double cantilever beam (TDCB) specimen. However, these two-component self-healing microcapsules are highly dependent on the curing agent, and there are some problems such as the high price of the curing agent, harmfulness to the human body, and low contact rate between the repairing agent and the curing agent [13,14]. Research has shown that self-healing coating, prepared by adding a certain number of microcapsules to the coating, has gradually increasingly been used in concrete coating, bonding coating, decorative coating [15], anti-corrosion coating, pavement coating, and in other fields [16,17]. Pooneh et al. [18] prepared a self-healing transparent coating for automobiles. Self-repairing microcapsules without catalyst were put into acrylic melamine transparent coating, and their scratch resistance was tested. The results showed that the particle size of the microcapsules affect its dispersion in the coating, and then affect the curing performance of the core repair agent. Schreiner et al. [19] designed a self-healing coating for metal corrosion protection or moisture sensitive substrate protection such as wood. The results showed that the self-healing coating with microcapsules had obvious advantages over the traditional protective coating. For the performance evaluation of microcapsule self-healing coating, the mechanical properties [20,21], corrosion-resistant properties [22], and repair efficiency are important [23]. Brown et al. [24] explored the fracture toughness and healing efficiency of epoxy resin substrate with self-healing microcapsules. They found that when the number of microcapsules increased, the fracture toughness of the matrix increased by 127% and the morphology of the fracture surface changed. Kumar et al. [25] prepared a variety of self-healing microcapsules with different core materials and added them to the coating system to explore the effects of the microcapsule addition method and coating interlayer drying time on interlayer adhesion. They found that when microcapsules were added in layers, the self-healing performance and adhesion of the coating were better. When the drying time between layers was short, the adhesion between layers was large, but the corrosion resistance would be reduced. At present, the core repair agents commonly used in self-repair coatings mainly include corrosion inhibition, epoxy, dry oil, siloxane, silicone ester, and isocyanate [26,27]. Shellac is a biodegradable natural resin. Its ethanol solution can be solidified into a film at room temperature without catalyst, and the gloss and toughness of the film are high, which is non-toxic and harmless to human body [28]. Therefore, choosing shellac as the core repair agent of self-healing coating has great advantages.

The modification of shellac is of great significance because of its brittleness and poor water and heat resistance. Therefore, this paper selected rosin to modify the aging resistance of shellac and explored the optimal ratio of rosin and shellac in the core material [29]. Microcapsules of a shellac–rosin mixture coated with melamine formaldehyde (MF)resin were prepared, and the preparation process parameters were improved. Then, the prepared self-healing microcapsules were mixed into the waterborne coating to explore the effect of the self-healing microcapsules on the elongation at break of the paint film samples into three modes (i.e., no cracks, when cracks occur, and after repairing), and the elongation at break was used to calculate the repair rate of the coating. The purpose of this paper was to explore the effects of different hydrophilic lipophilic balance (HLB) values on the performance of microcapsules and the self-healing performance of waterborne coatings with microcapsules and to obtain the comprehensive optimal self-healing waterborne coatings. This paper provides a basis for the performance evaluation of waterborne coatings with self-healing microcapsules in the future.

## 2. Materials and Methods

### 2.1. Experimental Materials

The details of the raw material information required for the tests are shown in Table 1. The waterborne coating comes from Dulux Co., Ltd., Shanghai, China and its main components were waterborne acrylic acid copolymer dispersion, matting agent, additive, and water.

### 2.2. Preparation Method of Modified Shellac Film

A 1.0 g shellac ethanol solution (25% mass ratio) and a 1.0 g mixture solution (25% mass ratio) with rosin and shellac at a mass ratio of 1:1 were coated on a glass plate. After being dried at room temperature for 20 min, it was moved into a 50 °C drying oven, heated until the quality did not change, and then taken out for subsequent aging resistance tests.

### 2.3. Preparation Method of Microcapsules

According to the relevant literature [30] on microcapsule preparation technology, four factors that can affect the preparation of microcapsules were determined, and the orthogonal test of 4 factors and 3 levels was carried out. As shown in Table 2 and Table 3, MF-coated shellac microcapsules with different morphologies and properties were prepared at 3 levels of the HLB value of emulsifier, the type of solvent (*W_ethanol_*:*W_distilled water_*), the mass ratio of shellac to rosin (*W_shellac_*:*W_rosin_*), and the rate of rotating. Among the 4 factors, the factor that had the greatest impact on the comprehensive properties of microcapsules and the best preparation scheme of microcapsules were explored. A single factor experiment was carried out for the most influential factor to further explore the relationship mechanism between HLB value and the performance of microcapsules.

The preparation process of microcapsules was mainly divided into three parts. Firstly, the 37.0% formaldehyde solution and melamine were massaged into a beaker with a molar ratio of 3.5:1, and then 30 mL of distilled water was added. After adjusting the pH value of the mixture to 8–9 with triethanolamine, the beaker was put into a 70 °C constant temperature water bath and stirred at 600 rpm for 30 min to obtain a water-soluble hydroxymethyl melamine mixture. Secondly, after the required emulsifier was weighed, it was added dropwise into the beaker together with 78.9 mL of distilled water and fully stirred and mixed. Then, the modified core material shellac liquid was added dropwise into the beaker. After mixing, it was put into a constant temperature water bath at 60 °C, and at a certain speed, the stable core material emulsion was obtained by stirring 60 min. Finally, the melamine–formaldehyde prepolymer solution was slowly dripped into the core emulsion at a certain speed, and then the mixture was put into ultrasonic material emulsifying disperser (Shanghai Bilang Instrument Co., Ltd., Shanghai, China) to ultrasonic treatment for 15 min. After ultrasonic treatment, the mixture was transferred to the water bath pot. At a certain speed, citric acid monohydrate was used to adjust the pH value. When the pH value reached approximately 4.5, the water bath pot was slowly heated to 60 °C for 3 h. After the mixture was left to stand for 3 d, it was washed with distilled water and absolute ethanol many times, then filtered and dried. The obtained product was the microcapsule sample. The details of the dosage of each substance in orthogonal and single-factor tests are shown in Table 4. *P_S_* represents the mass fraction of Span-20 in the mixed emulsifier; *P_t_* represents the mass fraction of Tween-20 in the mixed emulsifier; the HLB value (*H*) of mixed emulsifier was calculated as follows [31]:

*H* = *P_S_* × 8.6 + *P_t_* × 16.7

### 2.4. Preparation Method of the Self-Healing Coating

The microcapsules prepared with the five different HLB values were added into the waterborne coating with mass fractions of 0%, 3.0%, 6.0%, 9.0%, 12.0%, and 15.0%, respectively, and stirred and mixed evenly. The mixture samples were coated on the glass plates, and exposed to air for 20 min. The coated glass plates were put into a 50 °C drying oven and cured until the quality did not change and then taken out. The coating samples on the glass plates were gently removed from the substrate for tensile strength tests.

### 2.5. Testing and Characterization

Four groups of two kinds of shellac films before and after modification were prepared and placed in an oven at 60 and 120 °C for aging experiments. Before and after aging, the SEGT-J color difference instrument which came from Zhuhai Tianchuang Instrument Co., Ltd., Zhuhai, China, was used to measure the chromaticity value of the two shellac films before and after modification, and then the color difference (ΔE*) was calculated. Before and after aging, the HG268 gloss meter (Shenzhen 3nh Technology Co., Ltd., Shenzhen, China) was used to measure the gloss of the two shellac films before and after modification. The effect of shellac modification on its solubility in ethanol solution before and after aging was characterized by measuring the content of insoluble solids. The 1.0 g shellac membrane was added into the 10.0 mL ethanol solution, stirred for 5 h, and then the solution was filtered under vacuum. The filter paper was dried to a constant weight at 70 °C, and the percentage of insoluble solids was calculated according to the weight of dried solids.

The Axio Scope A_1_ biological optical microscope (OM) offered by Carl Zeiss AG, Oberkochen, Germany, and a Quanta-200 scanning electron microscope (SEM), from FEI Company, Hillsboro, OR, USA, were used to observe the micromorphology of the microcapsules, and the SEM images of shellac microcapsules were analyzed by Nano Measurer software to calculate the particle size. A VERTEX 80 V Fourier transform infrared spectroscopy (FTIR), supplied by Shanghai Smio Analytical Instrument Co., Ltd., Shanghai, China, was used to analyze the chemical composition of the microcapsules. The coating rate of the microcapsules was characterized by the weighing method. After microcapsules of *m*_1_ g were fully ground, they were fully soaked in ethyl acetate for 24 h and then soaked in ethanol for 48 h. Then, they was washed and filtered with distilled water to obtain the mass of residual wall material. The residue was weighed and dried (*m*_2_). The coating rate (*c*) of the microcapsules was calculated as follows:

*c* = (*m*_1_ − *m*_2_)/*m*_1_ × 100%

The cracks were created on some of the prepared self-healing coating samples by using a scalpel blade with a length of 2 cm, parallel to the stretching direction, and a depth of approximately 100 µm. The AG-IC100KN precision electronic universal testing machine (Shimadzu Manufacturing Institute, Kyoto, Japan) was used to conduct the tensile tests on the self-healing coating samples in three modes (before, at, and 24 h after the cracking), and the repair rate of the paint film was calculated through the elongation at break of the coating. *E_I_* is the elongation at break of the coating sample before the crack occurred, *E_S_* represents the elongation at break of the coating sample at the time of crack occurrence, and *E_H_* represents the elongation at break of the coating sample 24 h after the crack. The formula for calculating the repair rate *(ƞ)* of the coating is as follows [32]:

*ƞ* = (*E_H_* − *E_S_*)/(*E_I_* − *E_S_*) × 100%

All the tests were repeated four times with the error less than 5.0%.

## 3. Results and Discussion

### 3.1. Analysis of the Aging Resistance of Modified Shellac for the Microcapsule Core Material

#### 3.1.1. Analysis of the Optical Results

**Figure 1:**
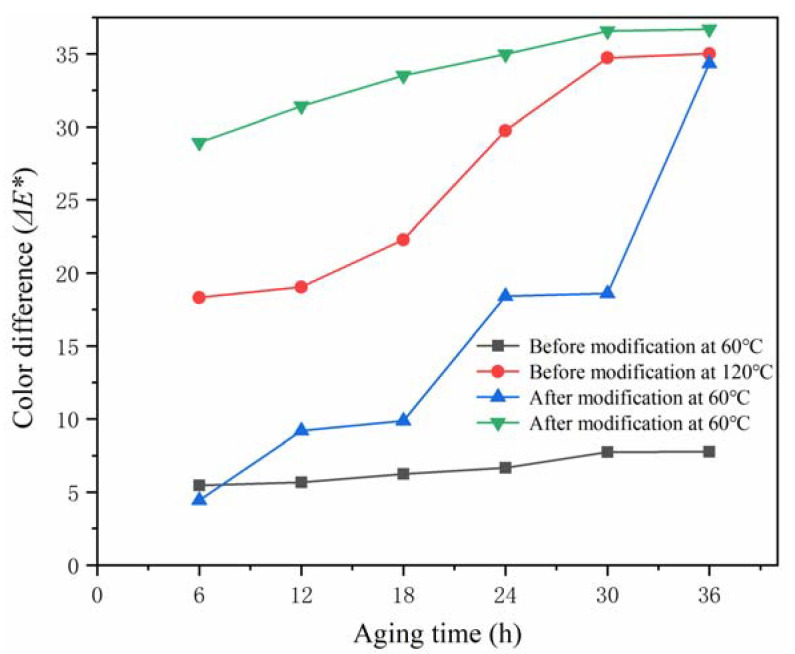
Changes in the color difference values of the shellac before and after modification.

The changes in the color difference (ΔE*) [33] and gloss of the shellac before and after modification are shown in Figure 1 and Table 5. In the aging process, the color difference of the modified shellac and the modified shellac rosin mixture increased, and the color difference of the modified core material was large. At the incident angle of 60 °, the gloss of the modified shellac rosin mixture was 113.45%, which was higher than that of the modified shellac. In the first aging cycle, the gloss of shellac decreased rapidly before and after modification, and decreased slowly when the aging time was 12–30 h. With the extension of aging time, the gloss of the shellac before and after modification decreased, which was due to the volatilization of ethanol, the overflow of small gas molecules, and the generation of bubbles on the film surface. With the increase in time, the bubbles broke, the surface reflection changed from a mirror reflection to a diffuse reflection, and the gloss decreased.

#### 3.1.2. Analysis of Insoluble Matter Content in Ethanol before and after Modification and Aging

**Figure 2:**
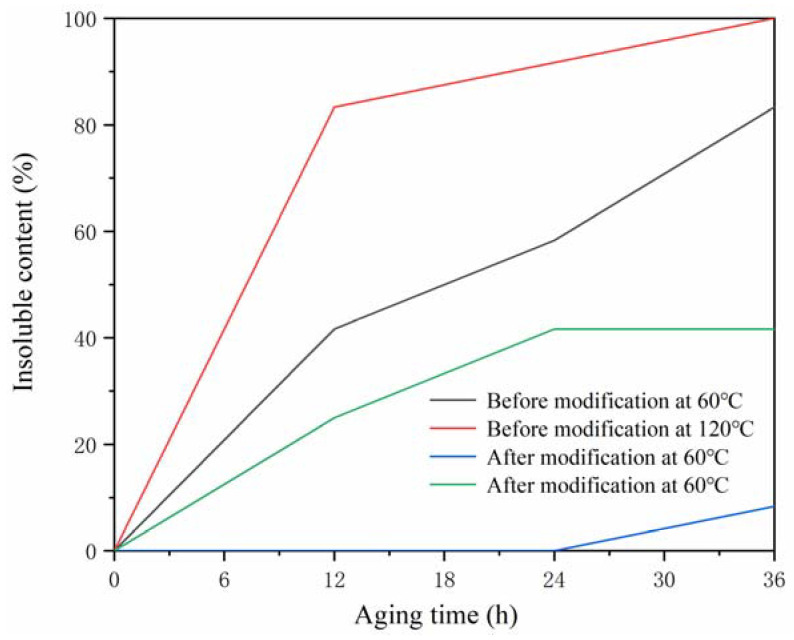
Changes in the insoluble matter content of shellac in ethanol solution before and after modification.

The core material shellac was modified with rosin to improve the hydrophilicity and aging resistance of the core material. It has been reported that the aging phenomenon of natural shellac is mainly manifested in its reduced solubility in ethanol [34]. Figure 2 shows the changes in the insoluble content of the core material in the ethanol solution before and after modification under high-temperature environments of 60 and 120 °C. With the increase in aging time, the content of the insoluble matter of the core material in the ethanol solution also increased. At 60 °C, after only 36 h of aging of the core material before modification, the content of insoluble matter in the ethanol solution exceeded 83.00%, while the content of insoluble matter in the ethanol solution for the modified core material was only 8.30%. At 120 °C, the solubility of the core material before modification was completely lost after aging for 36 h, and the content of insoluble matter in the ethanol solution of the modified core material was 41.67% at this time. It can be seen that rosin had a certain hindering effect on the aging of shellac.

### 3.2. Analysis of Orthogonal Test Results of Microcapsules

#### 3.2.1. Morphology Analysis of Microcapsules

**Figure 3:**
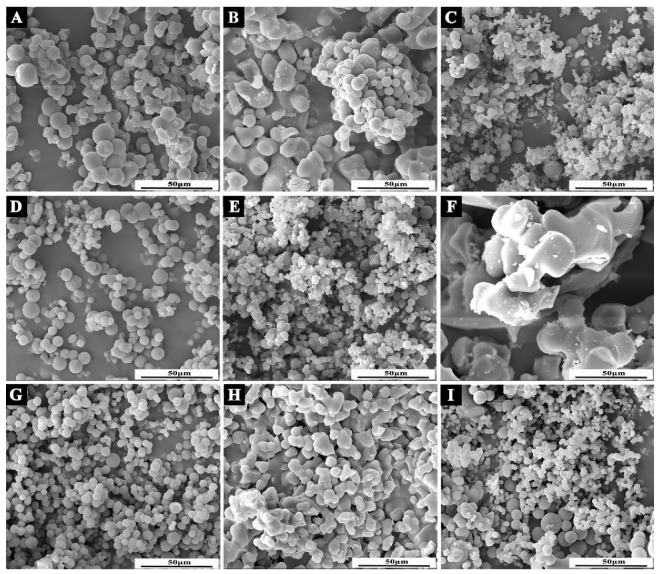
SEM of the microcapsules under orthogonal test parameters: (**A**) sample #1; (**B**) sample #2; (**C**) sample #3; (**D**) sample #4; (**E**) sample #5; (**F**) sample #6; (**G**) sample #7; (**H**) sample #8; (**I**) sample #9.

**Figure 4:**
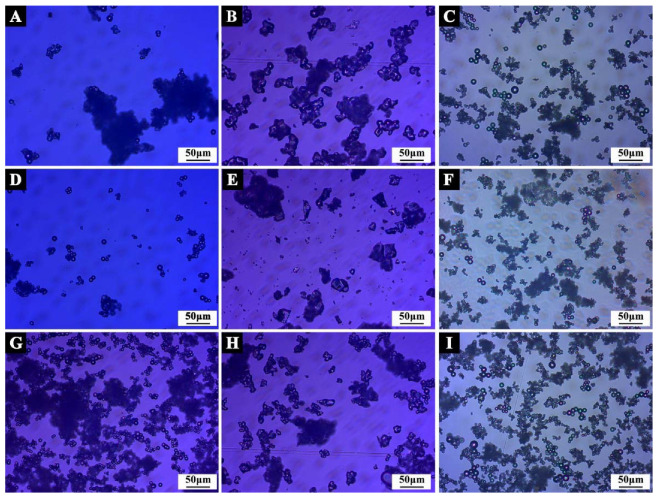
OM of microcapsules under orthogonal test parameters: (**A**) sample #1; (**B**) sample #2; (**C**) sample #3; (**D**) sample #4; (**E**) sample #5; (**F**) sample #6; (**G**) sample #7; (**H**) sample #8; (**I**) sample #9.

The micromorphology of microcapsules under orthogonal test parameters are shown in Figure 3 and Figure 4. In the process of microcapsule emulsification, the stability of core material in the emulsification system would affect the morphology of microcapsule. It can be seen that the morphology of microcapsule samples #6 and #8 (Figure 3F,H) with unmodified core material was irregular particles, forming obvious agglomeration, and the preparation effect of microcapsules was not ideal. In sample #1 (Figure 3A), ethanol was used as the solvent, and an obvious spherical shape could be observed, but the microcapsules adhered to each other, resulting in a large amount of agglomeration. Sample #2 (Figure 3B) had relatively few spherical microcapsules, poor dispersion, a serious agglomeration problem, and an unsatisfactory preparation effect. A large number of micro-spheres adhered to each other in sample #3 (Figure 3C), which may be due to the rapid rotation speed, excessive shear force and the rupture of microcapsules during the preparation process. Sample #4 (Figure 3D) was a regular sphere with smooth surface, good dispersion, and relatively uniform particle size. The preparation was relatively successful. Regular spheres could be observed in sample #5 (Figure 3E), but the adhesion was obvious. Flocs were attached to the surface of microcapsules, and the agglomeration was serious. The particles in sample #7 (Figure 3G) were smooth and had regular spheres with good dispersion; agglomeration basically disappeared and there was a good preparation effect. Sample #9 (Figure 3I) was a regular sphere, but the particle sizes were different, and there was little agglomeration.

When the light propagated in different media, a diffraction ring formed at the interface. In the OM picture (Figure 4), it can be clearly seen that there were two different media in the successfully prepared microcapsule, and the internal bright spot represented the presence of a core repair agent. Combined with the SEM and OM images, it can be seen that sample #7 was successfully prepared, and it had the best microstructure.

#### 3.2.2. Chemical Composition Analysis of the Microcapsules

**Figure 5:**
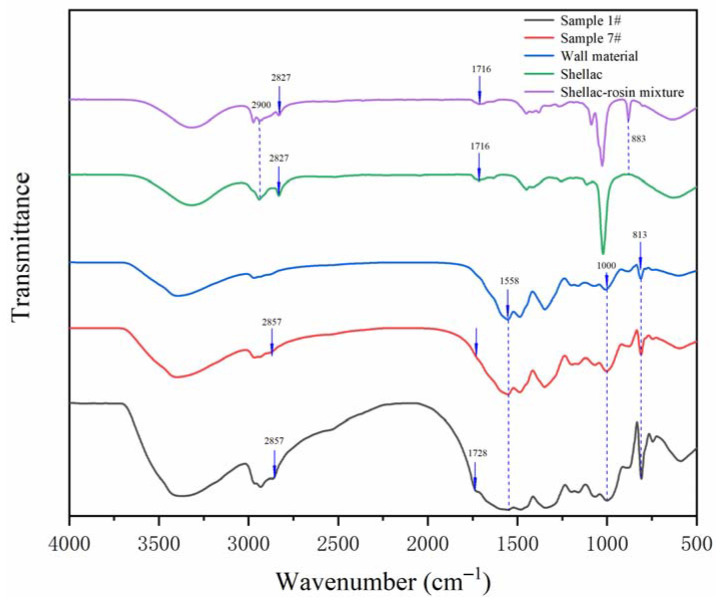
The FTIR of wall material, core material, and microcapsules in the orthogonal experiments.

The FTIR of the wall material, core material, and microcapsule samples are shown in Figure 5. By observing the infrared curves of shellac and the shellac–rosin mixture, it can be found that the peak near 2900 cm^−1^ describes the stretching vibration of the –CH_2_ group, and the peak widens after mixing with rosin. Compared with the shellac curve, the peak at 883 cm^−1^ disappeared. Comparing the microcapsule sample curve and the wall material curve, the triazine ring bending vibration absorption peak appeared at 813 cm^−1^ in the wall material MF resin. The stretching vibration absorption peak of N–H was approximately 1558 cm^−1^, and the 1000 cm^−1^ was the absorption peak of C–O. These peaks can also be observed on the curves of microcapsule samples. At the same time, it can be observed in the curves of the core material and microcapsule samples that 2827 and 2857 cm^−1^ were the characteristic peaks of –CH_3_, and the wide peak at 1716 cm^−1^ was the stretching vibration of C=O [35], indicating that shellac and shellac–rosin composite as core material were successfully coated by MF resin, and the microcapsules were successfully prepared.

#### 3.2.3. Analysis of Microcapsule Yield and Coating Rate

The yield of microcapsules is an important parameter in the process of microcapsule preparation. It is of great significance to produce high-yield microcapsules with less raw materials. Table 6 shows the range and variance results of microcapsule yield in orthogonal tests. The yield of sample #3 reached a maximum of 5.11 g. According to the variance results, it was found that the HLB value of emulsifier was the most influential factor on the yield of microcapsules, followed by the rate of rotating. The influence of the five factors was not significant. Combined with the range results, the better process parameters for preparing MF resin-coated shellac microcapsules were as follows: the HLB value of emulsifier was 12.65, *W_ethanol_*:*W_distilled water_* = 1:1 in solvent, *W_shellac_*:*W_rosin_* = 1:1 in core material, and the rate of rotating was 600 rpm.

Coating rate refers to the proportion of core repair agent in the total mass of microcapsules. The coating rate of core material in microcapsule is the key to its repair effect and an important index for evaluating the performance of microcapsules. The range and variance results of the coating rate in the orthogonal test are shown in Table 7. The variance results showed that the HLB value of the emulsifier had a significant effect on the coating rate of microcapsules, followed by the ratio of shellac to rosin in the core material. According to the range results, the better process parameters for preparing MF resin-coated shellac microcapsules were as follows: the HLB value of emulsifier was 12.65, *W_ethanol_*:*W_distilled water_* = 1:0 in solvent, *W_shellac_*:*W_rosin_* = 1:1 in core material, and the rate of rotating was 600 rpm.

Based on the results of orthogonal tests, the HLB value of the emulsifier had the greatest influence on the yield and coating rate of the microcapsules. Among them, the yield results showed that the optimal level was the *W_ethanol_*:*W_distilled water_* = 1:1 in solvent, *W_shellac_*:*W_rosin_* = 1:1 in the core material, and rotating rate of 600 rpm. The results of the coating rate showed that the optimal level was *W_ethanol_*:*W_distilled water_* = 1:0 in solvent, *W_shellac_*:*W_rosin_* = 1:1 in core material, and the rotating rate of 600 rpm. Combined with the optimal micromorphology of the sample #7 microcapsule, the preparation process parameters were *W_ethanol_*:*W_distilled water_* = 1:0 and the *W_shellac_*:*W_rosin_* = 1:1. Finally, the optimal parameter level was determined as *W_ethanol_*:*W_distilled water_* = 1:0 in solvent, *W_shellac_*: *W_rosin_* = 1:1 in core material, and the rotating rate of 600 rpm. On this basis, in order to explore the specific impact of the HLB value on the performance of the microcapsules, the HLB value of the emulsifier was taken as a single-factor variable, and other parameters were set as the better level of orthogonal experimental results.

### 3.3. Influence of the HLB Value on the Microcapsules

#### 3.3.1. Morphology Analysis of the Microcapsules

**Figure 6:**
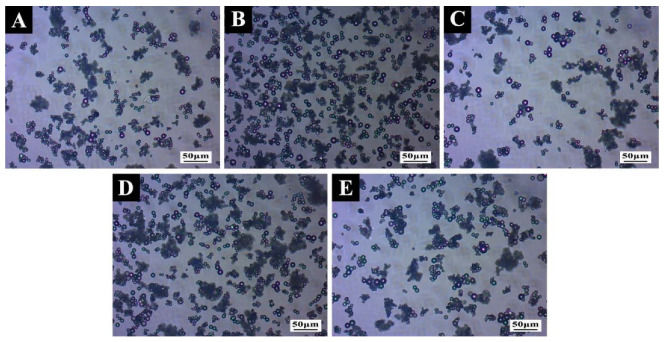
OM of the microcapsules under a single-factor test: (**A**) sample #10; (**B**) sample #11; (**C**) sample #12; (**D**) sample #13; (**E**) sample #14.

**Figure 7:**
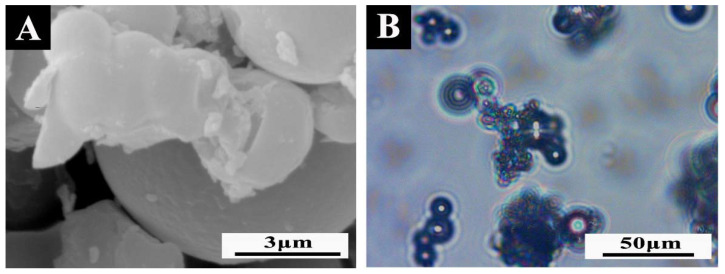
(**A**) SEM and (**B**) OM of damaged microcapsules.

The micromorphologies of damaged microcapsules and intact microcapsules are shown in Figure 6 and Figure 7. It can be observed from Figure 6 that the prepared shellac–rosin composite microcapsule obviously had two substances: the dark part of the outer ring was the wall material component, and the bright spot of the inner ring was the core material. According to the morphology of the damaged microcapsule in Figure 7, it can be clearly seen that there was an outflow of core material. Therefore, the prepared microcapsule had an obvious shell–core structure, and the core material was coated successfully.

**Figure 8:**
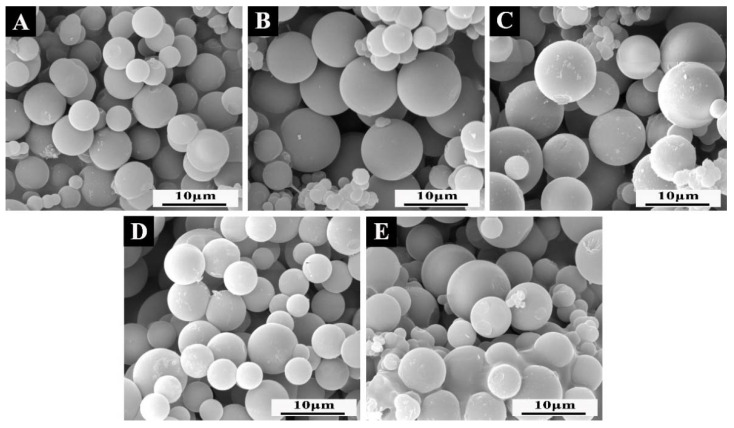
The SEM morphology of microcapsules: (**A**) sample #10; (**B**) sample #11; (**C**) sample #12; (**D**) sample #13; (**E**) sample #14.

**Figure 9:**
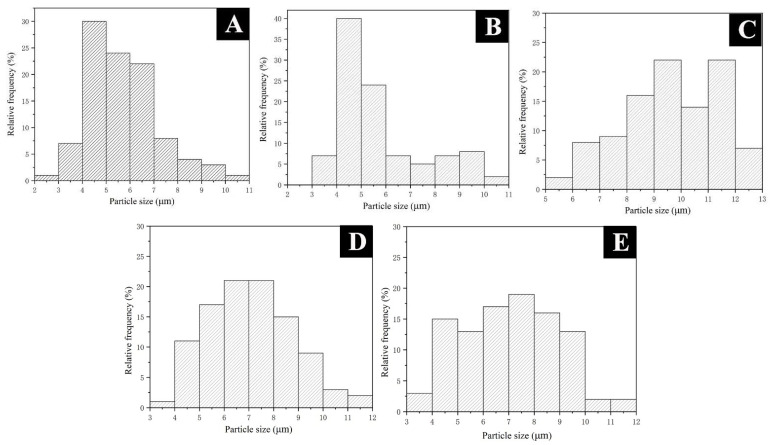
Particle size distribution of microcapsules: (**A**) sample #10; (**B**) sample #11; (**C**) sample #12; (**D**) sample #13; (**E**) sample #14.

In the preparation of microcapsules, the stability of the emulsion will affect the formation of microcapsules. The stability of the emulsion is closely related to the HLB value of the emulsifier. The SEM and particle size distribution images of microcapsules prepared under different HLB values of emulsifier are shown in Figure 8 and Figure 9. When the HLB value was 8.6 (Figure 8C), the microcapsules were slightly agglomerated, but the surface was rough, and the flocs were attached. The particle size of the microcapsules was 6–12 μm, with an average particle size of 9.7 μm. The particle size was too large and uneven. When the HLB value was 11.3 (Figure 8D), it could be clearly observed that the surface of the microcapsules was relatively smooth, with a small number of rough particles and relatively dispersed. The particle size of the microcapsules was 4–11 μm, with an average particle size of 7.10 μm. But the distribution was wide and different in size. When the HLB value increased to 12.65 (Figure 8A), the microcapsule had a smooth surface, good dispersion, and particle size of 3–10 μm with an average particle size of 5.72 μm. More than 75.0% of the particle size was distributed in 4–7 μm with a narrow distribution and uniform particle size. When the HLB value continued to increase to 14 (Figure 8E), the surface of the microcapsules was basically smooth, but the adhesion phenomenon was serious, and a large amount of agglomeration occurred, with an average particle size of 7.02 μm and uneven distribution. When the HLB value increased to 16.7 (Figure 8B), the surface of microcapsules was smooth, there was slight agglomeration, the particle size distribution was 3–10 μm, the distribution was relatively uniform, and the average particle size was 5.76 μm.

Based on the above analysis, with the increase in the HLB value of the emulsifier, the microscopic properties of the microcapsules basically showed a trend of first increasing and then decreasing. When the HLB value was 12.56, the prepared microcapsules with MF resin-coated shellac–rosin mixture were evenly distributed, with a small particle size, smooth surface, and good dispersion.

#### 3.3.2. Chemical Composition Analysis of Microcapsules

**Figure 10:**
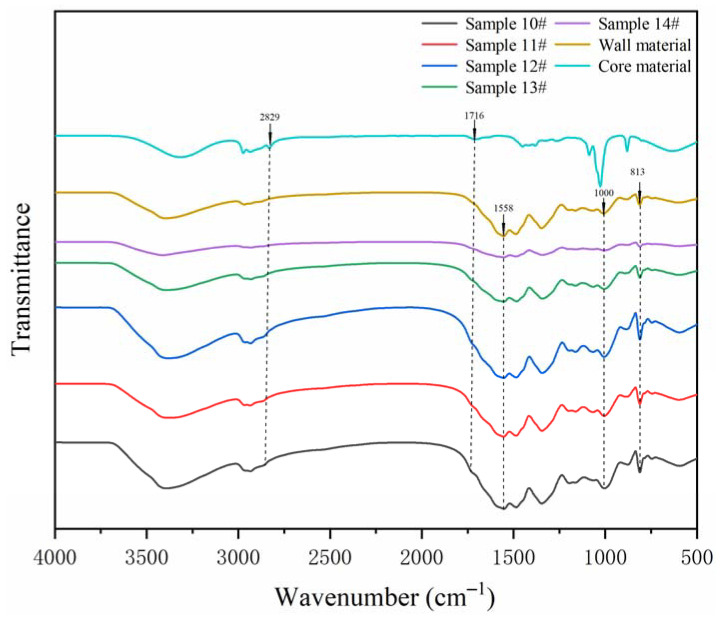
The FTIR of the microcapsules in single-factor tests.

The FTIR of microcapsule samples prepared by single-factor test is shown in Figure 10. The curve trend of microcapsules prepared with five different HLB values was basically the same, indicating that the composition of these five microcapsules was the same. The triazine ring bending vibration absorption peak appears at 813 cm^−1^ in the wall material. The stretching vibration absorption peak of N–H was approximately 1558 cm^−1^. The 1000 cm^−1^ was the absorption peak of C–O, and the corresponding peak also appeared in the five microcapsule samples. At the same time, it can be observed in the curves of core material (shellac–rosin composite) and microcapsule that the 2827 and 2857 cm^−1^ were the characteristic peaks of –CH_3_. The wide peak at 1716 cm^−1^ was the stretching vibration of C=O, indicating that the microcapsule of shellac–rosin composite coated with MF resin was successfully prepared, and the chemical composition did not change.

#### 3.3.3. Analysis of the Yield and Coating Rate

The results of yield and coating rate of the microcapsules prepared with different HLB values are shown in Table 8. With the increasing HLB value of emulsifier, the yield and coating rate of the microcapsules showed the same law, which basically increased first and then decreased. When the HLB value of emulsifier was 12.65, the maximum yield of microcapsule was 17.96 g, and the coating rate of microcapsule was also the highest, which was 26.44%. This may be because when the HLB value was 12.56, the dispersion between emulsified particles was the largest, and the emulsion was the most stable in the emulsification process. The lipophilic group of the emulsifier was adsorbed onto the surface of shellac–rosin composite droplets, and the hydrophilic group attracted the active group of melamine–formaldehyde prepolymer close to it, which was successfully deposited on the surface of small droplets of core material under the initiation of H^+^. Taking the HLB value as a single factor for analysis of variance (Table 9), according to the yield, it was concluded that F_ratio_ = 0.032, F_critical value_ = 5.317, and F_ratio_ < F_critical value_, so the HLB value had no significant impact on the yield. At the same time, according to the coating rate, the F_ratio_ = 17.921, while the F_critical value_ = 5.318, and the F_ratio_ > F_critical value_. Therefore, the HLB value had a significant effect on the coating rate of microcapsules.

### 3.4. Analysis of the Self-Healing Performance of Waterborne Coatings

**Figure 11:**
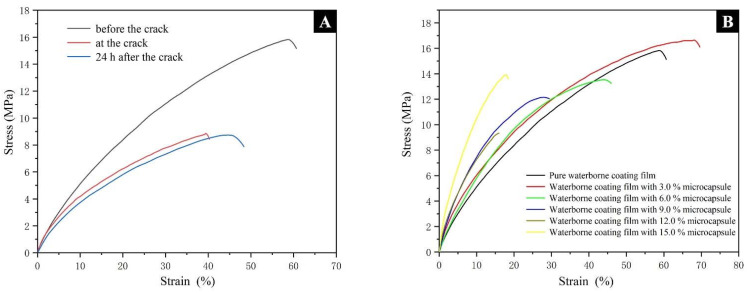
Stress–strain curves: (**A**) the pure waterborne coating before, at, and 24 h after the cracking; (**B**) waterborne coating containing microcapsules with different mass fractions.

The stress–strain curves of the pure waterborne coating before, at, and 24 h after the crack are shown in Figure 11A. The tensile properties of the pure waterborne coating decreased significantly when the crack occurred, and the tensile properties of the coating did not improve 24 h after the crack occurred. It can be seen that the defects of the waterborne coating would lead to a decline in the mechanical properties of the coating. A coating with self-healing function was very needed. The elongation at break results in coatings in three modes (i.e., no cracks, when cracks occur, and after repairing) with different kinds of microcapsule samples as shown in Table 10. When the additional number of microcapsules was the same, the elongation at break of the waterborne coating added with microcapsule sample #10 and #13 was relatively high, and the toughness of the coating was relatively good. However, with the increase in the number of microcapsules, the elongation at break of the waterborne coating added in microcapsule sample #10 decreased from 65.58% to 18.08%. With the increase in microcapsule content, the elongation at break of the water-based coating of microcapsule sample #10 decreased from 65.58% to 18.08%, and the amplitude was relatively small, while the elongation at break of the waterborne coating added to microcapsule sample #13 decreased from 90.49% to 9.15%, with a larger decrease. With the increase in the content of microcapsules, the tensile strength of the waterborne coating basically showed a downward trend (Figure 11B). This was because when the load of microcapsules in the coating increased, it would lead to the aggregation of microcapsules, resulting in more bubbles in the aqueous acrylic acid matrix. The defects in the coating film led to a reduction in the overall mechanical properties, while the elongation at break of the waterborne coating added with a 3.0% mass fraction in microcapsule sample #10 increased to 65.58%, maintaining the original mechanical properties.

While maintaining the mechanical properties of the coating, the repair rate of the coating is an important index to evaluate the self-healing performance of the coating. The repair rate of waterborne coating with microcapsules prepared by different HLB values are shown in Table 11. When the load of microcapsule increased from 3.0% to 12.0%, the repair rate of the coating with microcapsule prepared with the HLB value of 12.65 (sample #10) increased from 43.79% to 81.17%. When the load of the microcapsule increased, the core healing agent was increased and filled to the crack; thus, the repair rate was improved. However, with the increasing load of microcapsules, the repair rate of waterborne coatings with different microcapsules increased first and then decreased. When the microcapsule load was constant, with the continuous increase in the HLB value, the repair rate of waterborne coating increased first and then decreased. Compared with the waterborne coating added with other microcapsules prepared with other HLB value, the waterborne coating added with microcapsules prepared with the HLB value of 12.65 had a greater improvement in the repair rate with the increase in microcapsule content. Therefore, in order to achieve a balance between maintaining the original mechanical properties of the coating and obtaining a higher self-healing effect, on the whole, the waterborne coating with microcapsules prepared with an HLB value of 12.65 had a better comprehensive effect.

## 4. Conclusions

The MF resin-coated shellac microcapsules were successfully prepared through the orthogonal experiment of 4 factors and 3 levels. The optimal parameter levels were determined as *W_ethanol_*:*W_distilled water_* = 1:0 in solvent, *W_shellac_*:*W_rosin_* = 1:1, and the rate of rotating of 600 rpm. The factor that had the greatest impact on the comprehensive performance of the microcapsules was the HLB value of the emulsifier. In the single-factor experiments, with the increasing HLB value of the emulsifier, the micro properties, coating rate, and yield of the microcapsules basically increased first and then decreased. When the HLB value was 12.56, the prepared MF resin-coated shellac–rosin mixture microcapsules had smaller particle sizes and better comprehensive performance. The microcapsules prepared with five different HLB values were added to the waterborne coating to prepare self-healing coating. The elongation at break of the waterborne coating added with microcapsules obtained at HLB of 12.56 at 3.0% mass fraction was increased to 65.58%, which maintained the original mechanical properties. At this time, the repair rate of the coating was 43.79%, which had a relatively obvious repair effect. When the microcapsule load was constant, with the continuous increase in the HLB value, the repair rate of the waterborne coating increased first and then decreased. This paper provided a technical basis for the application of self-healing microcapsules in waterborne coatings.

## Figures and Tables

**Table 1:** List of raw material information for the experiments.

Experimental Materials	Molecular Mass (g/mol)	CAS	Manufacturer
37.0% Formaldehyde	30.03	50-00-0	Xi’an Tianmao Chemical Co., Ltd., Xi’an, China
Melamine	126.15	108-78-1	Shandong Yousuo Chemical Technology Co., Ltd., Linyi, China
Triethanolamine	149.19	102-71-6	Guangzhou Jiale Chemical Co., Ltd., Guangzhou, China
Span-20	346.459	1338-39-2	Shandong Yousuo Chemical Technology Co., Ltd., Linyi, China
Tween-20	1227.5	9005-64-5	Shandong Yousuo Chemical Technology Co., Ltd., Linyi, China
Shellac	964–1100	9000-59-3	Shanghai Yuyan Building Materials Co., Ltd., Shanghai, China
Rosin	302.46	8050-09-7	Suzhou Guyue Musical Instrument Co., Ltd., Suzhou, China
Citric acid monohydrate	210.14	5949-29-1	Nanjing Quanlong Biotechnology Co., Ltd., Nanjing, China
Anhydrous ethanol	46.07	64-17-5	Wuxi Jingke Chemical Co., Ltd., Wuxi, China
Ethyl acetate	88.11	141-78-6	Xi’an Tianmao Chemical Co., Ltd., Xi’an, China

**Table 2:** The factors and levels of the orthogonal experiment.

Level	HLB Value of Emulsifier	Solvent (*W_ethanol_:W_distilled water_*)	Core Materials (*W_shellac_:W_rosin_*)	Rate of Rotating (rpm)
1	12.65	1:0	1:0	600
2	15.08	2:1	1.5:1	800
3	10.22	1:1	1:1	1000

**Table 3:** The arrangement of orthogonal experiment.

Sample	HLB Value of Emulsifier	Solvent (*W_ethanol_:W_distilled water_*)	Core Materials (*W_shellac_:W_rosin_*)	Rate of Rotating (rpm)
#1	12.65	1:0	1:0	600
#2	12.65	2:1	1.5:1	800
#3	12.65	1:1	1:1	1000
#4	15.08	1:0	1.5:1	1000
#5	15.08	2:1	1:1	600
#6	15.08	1:1	1:0	800
#7	10.22	1:0	1:1	800
#8	10.22	2:1	1:0	1000
#9	10.22	1:1	1.5:1	600

**Table 4:** A detailed list of the amount of material in the orthogonal and the single-factor experiments.

Experiment	Sample	Melamine (g)	37.0% Formaldehyde (g)	Shellac (g)	Rosin (g)	Span-20 (g)	Tween-20 (g)	Ethanol (mL)	Distilled Water (mL)
Orthogonal experiment	#1	6	13.52	8.80	0	0.15	0.15	78.90	0
	#2	6	13.52	5.28	3.52	0.15	0.15	52.60	26.30
	#3	6	13.52	4.40	4.40	0.15	0.15	39.45	39.45
	#4	6	13.52	5.28	3.52	0.06	0.24	78.90	0
	#5	6	13.52	4.40	4.40	0.06	0.24	52.60	26.30
	#6	6	13.52	8.80	0	0.06	0.24	39.45	39.45
	#7	6	13.52	4.40	4.4	0.24	0.06	78.90	0
	#8	6	13.52	8.80	0	0.24	0.06	52.60	26.30
	#9	6	13.52	5.28	3.52	0.24	0.06	39.45	39.45
Single-factor experiment	#10	12	27.04	8.80	8.80	0.30	0.30	157.80	0
	#11	12	27.04	8.80	8.80	0	0.60	157.80	0
	#12	12	27.04	8.80	8.80	0.60	0	157.80	0
	#13	12	27.04	8.80	8.80	0.40	0.20	157.80	0
	#14	12	27.04	8.80	8.80	0.20	0.40	157.80	0

**Table 5:** Change in the gloss of the shellac before and after modification.

Sample Type	Aging Time (h)	Oven at 60 °C			Oven at 120 °C		
		20° Gloss (%)	60° Gloss (%)	85° Gloss (%)	20° Gloss (%)	60° Gloss (%)	85° Gloss (%)
Pure shellac before modification	0	128.40	107.00	87.93	128.40	107.00	87.93
	6.0	119.58	130.25	91.53	82.10	97.05	80.80
	12.0	97.40	118.15	86.73	85.80	90.88	79.55
	18.0	93.70	117.70	85.20	65.40	92.25	76.95
	24.0	82.60	114.95	84.15	38.58	88.78	79.88
	30.0	76.65	113.50	83.23	27.28	89.15	71.08
	36.0	61.65	91.23	78.50	24.73	88.05	66.50
Shellac–rosin mixture after modification	0	98.20	113.45	65.30	98.20	113.45	65.30
	6.0	57.98	92.90	91.13	76.88	85.95	71.68
	12.0	51.13	92.80	83.00	74.85	78.53	71.55
	18.0	36.40	87.58	75.13	39.78	77.35	67.25
	24.0	33.00	82.03	63.30	36.80	75.53	66.30
	30.0	23.53	57.50	56.73	23.08	70.20	65.53

**Table 6:** Range and variance results of yield of microcapsules.

	Sample	HLB Value of Emulsifier	Solvent (*W_ethanol_:W_distilled water_*)	Core Materials (*W_shellac_:W_rosin_*)	Rate of Rotating (rpm)	Yield (g)
Range	#1	12.65	1:0	1:0	600	4.78
	#2	12.65	2:1	1.5:1	800	4.50
	#3	12.65	1:1	1:1	1000	5.11
	#4	15.08	1:0	1.5:1	1000	4.05
	#5	15.08	2:1	1:1	600	4.55
	#6	15.08	1:1	1:0	800	4.25
	#7	10.22	1:0	1:1	800	4.12
	#8	10.22	2:1	1:0	1000	4.74
	#9	10.22	1:1	1.5:1	600	4.58
	Mean 1	4.797	4.317	4.590	4.637	
	Mean 2	4.283	4.597	4.377	4.290	
	Mean 3	4.480	4.647	4.593	4.633	
	*R*	0.514	0.330	0.216	0.347	
Variance	Sum of Squared Deviations	0.402	0.190	0.092	0.238	
	Degrees of Freedom	2	2	2	2	
	F_ratio_	4.370	2.065	1.000	2.587	
	F_critical value_	9.000	9.000	9.000	9.000	
	Significance					

**Table 7:** Range and variance results of the coating rate of microcapsules.

	Sample	HLB Value of Emulsifier	Solvent (*W_ethanol_:W_distilled water_*)	Core Materials (*W_shellac_:W_rosin_*)	Rate of Rotating (rpm)	Coating Rate (%)
Range	1#	12.65	1:0	1:0	600	23.0
	2#	12.65	2:1	1.5:1	800	15.0
	3#	12.65	1:1	1:1	1000	21.0
	4#	15.08	1:0	1.5:1	1000	14.0
	5#	15.08	2:1	1:1	600	19.0
	6#	15.08	1:1	1:0	800	13.0
	7#	10.22	1:0	1:1	800	12.0
	8#	10.22	2:1	1:0	1000	13.0
	9#	10.22	1:1	1.5:1	600	8.0
	Mean 1	19.667	16.333	16.333	16.667	
	Mean 2	15.333	15.667	12.333	13.333	
	Mean 3	11.000	14.000	17.333	16.000	
	*R*	8.667	2.333	5.000	3.334	
Variance	Sum of Squared Deviations	112.667	8.667	42	18.667	
	Degrees of Freedom	2	2	2	2	
	F_ratio_	13.000	1.000	4.846	2.154	
	F_critical value_	9.000	9.000	9.000	9.000	
	Significance	*				

* significantly difference.

**Table 8:** Results of yield and coating rate in single factor test.

Sample	HLB Value of Emulsifier	Yield (g)	Coating Rate (%)
#10	12.65	17.96	26.44
#11	16.7	6.89	15.56
#12	8.6	12.52	23.16
#13	11.3	9.89	17.89
#14	14.0	14.56	20.21

**Table 9:** Variance analysis of yield and coating rate in single factor test.

	Sum of Squared Deviations	Degrees of Freedom	F_ratio_	F_critical value_	Significance
Yield	0.33124	1	0.032	5.317	
Coating rate	187.14	1	17.921	5.318	*

* significantly difference.

**Table 10:** Elongation at break results of waterborne coatings with different microcapsules under three modes.

Microcapsule Type	Microcapsule Content (%)	Elongation at Break of Coating (%)		
		Before the Cracking	At the Cracking	24 h after Cracking
#10	0	58.14	40.07	43.77
	3.0	65.58	32.58	47.03
	6.0	44.60	21.59	31.47
	9.0	27.86	16.28	24.64
	12.0	21.57	13.67	20.08
	15.0	18.08	12.22	15.32
#11	3.0	7.42	4.00	5.39
	6.0	7.45	3.10	5.55
	9.0	7.00	2.85	5.84
	12.0	5.59	5.00	5.14
	15.0	6.75	2.01	4.37
#12	3.0	53.32	8.71	24.55
	6.0	35.00	7.21	13.84
	9.0	32.56	6.61	10.65
	12.0	29.46	3.65	7.40
	15.0	14.71	2.91	4.97
#13	3.0	90.49	37.97	63.34
	6.0	57.32	8.66	32.44
	9.0	29.30	7.02	19.85
	12.0	11.89	4.27	8.82
	15.0	9.15	4.34	7.92
#14	3.0	94.71	19.85	82.26
	6.0	26.35	9.06	22.14
	9.0	21.13	5.87	14.65
	12.0	20.52	3.74	6.12
	15.0	14.84	0.92	4.40

**Table 11:** Results of the repair rate of waterborne coatings with different microcapsules.

Microcapsule Content (%)	Repair Rate of Coating (%)				
	Microcapsule Sample #10	Microcapsule Sample #11	Microcapsule Sample #12	Microcapsule Sample #13	Microcapsule Sample #14
0	20.49	20.49	20.49	20.49	20.49
3.0	43.79	40.75	35.51	48.31	83.37
6.0	42.94	56.34	23.88	23.88	75.63
9.0	72.22	72.12	15.55	57.58	57.56
12.0	81.17	23.95	14.53	59.75	14.19
15.0	52.96	49.90	17.46	74.41	25.00

## Data Availability

Not applicable.
